# AKT Inhibitor Capivasertib as a Target for Osimertinib Combination Therapy in Lung Adenocarcinoma with *EGFR* Mutation

**DOI:** 10.3390/genes17091016

**Published:** 2026-08-27

**Authors:** Takuya Tokunaga, Yuka Ishihara, Aya Harada Takeda, Yuya Tomioka, Ayako Nagata, Mayuko Kato, Takayuki Suetsugu, Keiko Mizuno, Kazuhiro Ueda, Naohiko Seki

**Affiliations:** 1 Department of General Thoracic Surgery, Graduate School of Medical and Dental Sciences, Kagoshima University, Kagoshima 890-8520, Japan; k1933430@kadai.jp (T.T.); k7007579@kadai.jp (Y.I.); k2106983@kadai.jp (A.H.T.); k7433286@kadai.jp (K.U.); 2 Department of Pulmonary Medicine, Graduate School of Medical and Dental Sciences, Kagoshima University, Kagoshima 890-8520, Japan; k4829264@kadai.jp (Y.T.); k5090698@kadai.jp (T.S.); keim@m.kufm.kagoshima-u.ac.jp (K.M.); 3 Department of Breast and Thyroid Surgery, Graduate School of Medical and Dental Sciences, Kagoshima University, Kagoshima 890-8520, Japan; k8235150@kadai.jp; 4 Department of Functional Genomics, Graduate School of Medicine, Chiba University, Chiba 260-8670, Japan; mayukokato@chiba-u.jp

**Keywords:** *miR-126-3p*, molecular pathway, *EGFR* mutation, *AKT2*, osimertinib, capivasertib

## Abstract

**Background/Objective**: An analysis of microRNA (miRNA) expression signatures in lung adenocarcinoma (LUAD) harboring *EGFR* mutations revealed that multiple miRNAs, including *miR-126-3p*, were suppressed in cancer tissues. This study focused on *miR-126-3p* and aimed to identify therapeutic target genes regulated by *miR-126-3p* in LUAD harboring *EGFR* mutations and evaluate their potential for combination therapy with EGFR tyrosine kinase inhibitors. **Methods**: The antitumor function of *miR-126-3p* was analyzed by the ectopic expression of *miR-126-3p* into LUAD cells with *EGFR* mutation. The target genes of *miR-126-3p* were identified through an analysis of the TargetScan, Genecodis 4 and TCGA databases. The Chou–Talalay method was used to determine the potential synergistic effects of selected drug combinations. **Results**: The expression of *miR-126-3p* attenuated the malignant transformation of LUAD cells through targeting the “EGFR tyrosine kinase inhibitor resistance pathway”. We focused on *AKT2* among the genes involved in this pathway. Capivasertib was recently approved as the first inhibitor of oncogenic *AKT* signaling. Therefore, we investigated the effect of combination therapy comprising osimertinib and capivasertib on LUAD cell viability. Combination therapy synergistically reduced cell viability in both PC9 and H1975 cells, with combination index values of 0.61 and 0.14, respectively, at Fa = 0.5. **Conclusions**: Our miRNA-based analysis is an excellent strategy for identifying therapeutic targets that enhance the effects of EGFR inhibitors on LUAD.

## 1. Introduction

Approximately 2.5 million people worldwide are diagnosed with lung cancer each year. Lung cancer is the leading cause of death among malignant tumors, with more than 1.8 million deaths annually [1]. Histologically, the most common type is non-small cell lung cancer (NSCLC), accounting for approximately 85% of all lung cancer cases [2]. Previous cancer genome analyses have revealed several driver gene mutations involved in the development, malignancy, and drug resistance of NSCLC [3]. In recent years, various drugs that target these driver gene mutations have been developed; thus, testing for driver gene mutations is crucial for selecting appropriate targeted therapies and determining treatment strategies [4].

The prevalence of epidermal growth factor receptor (*EGFR*) gene mutations in NSCLC, especially lung adenocarcinoma (LUAD), is approximately 15–20% worldwide [5]. *EGFR* mutations are common in East Asians, including Japanese individuals, and are detected in approximately 30–50% of LUAD patients [6]. Activating *EGFR* mutations confer sensitivity to EGFR-TKIs. Currently, the main treatment for patients with advanced NSCLC harboring *EGFR* mutations is the use of EGFR tyrosine kinase inhibitors (TKIs) [7]. Since the introduction of the first-generation EGFR-TKI gefitinib in 2002, various EGFR-TKIs have been developed and have significantly improved the prognosis of lung cancer patients [8]. However, cancer cells have been reported to acquire drug resistance after the long-term use of EGFR-TKIs [9]. Osimertinib is a third-generation EGFR-TKI effective against the T790M mutation, but its effectiveness may decrease if other *EGFR* mutations arise during treatment; a typical example is the C797S mutation, and there are few effective drugs for cancer cells with this mutation [10].

Exploring the oncogenic RNA networks activated in *EGFR*-mutant LUAD cells may lead to the discovery of new therapeutic target molecules for LUAD. MicroRNAs (miRNAs) are short, single-stranded noncoding RNAs that each regulate the expression of numerous protein-coding genes within a cell [11]. Therefore, abnormalities in miRNA expression (upregulation or downregulation) may disrupt the RNA networks between miRNAs and protein-coding genes in cells [12]. The widespread use of RNA sequencing has made it easier to create miRNA signatures and identify abnormal miRNA expression in various types of cancers [13]. Numerous studies have demonstrated that aberrantly expressed miRNAs are intricately involved in the malignant transformation of cancer cells, including their development, metastasis, and drug resistance [14].

Based on miRNA expression signatures, we have been exploring the oncogenic RNA networks in cancer cells regulated by antitumor miRNAs [15,16]. To elucidate these networks within *EGFR*-mutant LUAD cells, we generated an miRNA expression signature using tissue from patients with *EGFR*-mutant LUAD [17]. According to this signature, *miR-126-3p* was significantly reduced in cancer compared with normal tissues.

In this study, we focused on *miR-126-3p* and investigated its regulated RNA networks in LUAD cells. Our in silico analysis revealed that 10 genes were involved in the “EGFR tyrosine kinase inhibitor resistance pathway”, one of which was *AKT2*, and they are closely involved in the molecular pathogenesis of *EGFR*-mutant LUAD. In 2024, capivasertib was designated the first approved inhibitor of oncogenic *AKT* signaling [18]. Therefore, we investigated the effect of combination therapy consisting of an EGFR inhibitor (osimertinib) and AKT inhibitor (capivasertib) on *EGFR*-mutant LUAD cells. The combination of the two drugs synergistically suppressed the viability of cancer cells compared with monotherapy.

The miRNA analysis used for LUAD in this study is an effective strategy for exploring the oncogenic pathways associated with this disease. Furthermore, identifying target molecules regulated by miRNAs will provide useful information for developing new therapeutic regimens for lung cancer.

## 2. Materials and Methods

### 2.1. LUAD Cell Lines Harboring EGFR Mutation

Human *EGFR*-mutant LUAD cell lines PC9 and H1975 were obtained from RIKEN BRC (Tsukuba, Japan) and the American Type Culture Collection (Manassas, VA, USA), respectively. PC9 cells harbor an *EGFR* exon 19 deletion, whereas H1975 cells harbor *EGFR* L858R and T790M mutations. Two cell lines were cultured in RPMI-1640 supplemented with 10% fetal bovine serum at 37 °C in a humidified atmosphere containing 5% CO_2_.

### 2.2. Bioinformatic Analysis

LUAD gene expression data were obtained from TCGA (https://www.cancer.gov/tcga, accessed on 20 December 2025), the GDC Data Portal (https://portal.gdc.cancer.gov/, accessed on 20 December 2025), and FireBrowse (http://firebrowse.org/, accessed on 20 December 2025). Gene expression levels were analyzed using log2-transformed RSEM values [log2 (RSEM + 1)]. Expression levels were compared between normal lung tissues (*n* = 59) and LUAD tissues (*n* = 516) using Welch’s two-sample *t*-test. Survival data were retrieved from OncoLnc (https://www.oncolnc.org/, accessed on 20 December 2025) and cBioPortal (https://www.cbioportal.org/, accessed on 20 December 2025). Patients were stratified into high- and low-expression groups using the median expression level of each miRNA or gene as the cutoff. To evaluate five-year overall survival, follow-up was administratively censored at 60 months, with all patients retained in the analysis. Kaplan–Meier survival curves were generated, and differences between the high- and low-expression groups were assessed using the log-rank test. For the analyses of the 10 candidate genes, *p*-values for both gene expression and survival analyses were adjusted separately for multiple comparisons using the Benjamini–Hochberg false discovery rate (FDR) method.

### 2.3. Prediction of miR-126-3p Oncogenic Pathway and Target Genes

The search for the candidate target genes of *miR-126-3p* used the TargetScanHuman database, including all predicted targets regardless of the conservation status of the miR-126-3p-binding sites. The identified genes were subjected to functional enrichment analysis using GeneCodis 4 to identify pathways associated with TKI resistance [19]. The candidate genes were further filtered using TCGA expression data to identify those upregulated in LUAD compared with normal lung tissues.

### 2.4. Transfection of miRNA Mimics into LUAD Cells

PC9 and H1975 cells were seeded in 6-well plates at a density of 4 × 10^4^ cells/mL and transiently transfected with an *miR-126-5p* or *miR-126-3p* precursor at a final concentration of 10 nM using Lipofectamine RNAiMAX. Mock-transfected cells received the transfection reagent without an miRNA precursor, whereas negative control cells were transfected with a negative control miRNA at 10 nM. Cells were harvested 72 h after transfection for RNA and protein analyses. For cell viability analysis, PC9 and H1975 cells were seeded in 96-well plates at a density of 2.0 × 10^3^ cells per well and transfected under the same conditions. Cell viability was assessed 72 h after transfection using the Cell Counting Kit-8 (CCK-8) assay and expressed relative to mock-transfected cells, which were set to 100%. Experiments were performed in at least three independent biological replicates. Detailed information on the reagents used is provided in Table S1.

### 2.5. Quantitative PCR Analysis

Total RNA was extracted from PC9 and H1975 cells 72 h after miRNA transfection and reverse-transcribed into cDNA. Quantitative real-time PCR was performed using SYBR Green chemistry on a QuantStudio 3 Real-Time PCR System. Relative gene expression was calculated using the 2^−ΔΔCt^ method, normalized to *GAPDH*, and expressed relative to mock-transfected cells. Three independent biological experiments were performed, with each sample analyzed in technical triplicate. Detailed information on the reagents and primer sequences is provided in Table S1.

### 2.6. Western Blot Analysis

Proteins were extracted from PC9 and H1975 cells 72 h after miRNA transfection using RIPA lysis buffer supplemented with protease and phosphatase inhibitors. Equal amounts of protein (10 µg per lane) were separated by SDS-PAGE and transferred to PVDF membranes using a Trans-Blot Turbo Transfer System with the manufacturer’s preset transfer program. After blocking, the membranes were incubated with the appropriate primary and secondary antibodies, and protein signals were detected by chemiluminescence. GAPDH was used as an internal control. Western blot experiments were performed in three independent biological replicates. Detailed information on the antibodies and reagents used is provided in Table S1.

### 2.7. Anticancer Effects of EGFR Inhibitor and AKT Inhibitor in LUAD Cells with EGFR Mutations

PC9 and H1975 cells were seeded in 96-well plates at a density of 2.0 × 10^3^ cells per well and incubated for 24 h before drug treatment. Cells were treated with osimertinib or capivasertib at 0.001, 0.01, 0.03, 0.1, 0.3, 1, 3, 10, 30, and 100 µM for 72 h. Both drugs were dissolved in DMSO, and vehicle-treated cells containing 0.1% DMSO served as controls.

Cell viability was assessed using CCK-8. After the addition of the CCK-8 reagent, absorbance at 450 nm was measured at 1 h intervals using a microplate reader. The first time point at which the mean absorbance of the vehicle-treated control wells exceeded 1.0 was used for analysis. Cell viability was expressed relative to vehicle-treated control cells. IC50 values were calculated using R (version 4.5.0) by fitting four-parameter log-logistic dose–response curves.

For combination experiments, osimertinib and capivasertib were administered at a fixed molar ratio of 1:10. Drug interactions were evaluated using the Chou–Talalay combination index (CI) method. The fraction affected (Fa) was calculated as 1 − cell viability/100, and CI values were calculated using the mutually exclusive model. CI values <1, =1, and >1 indicated synergistic, additive, and antagonistic effects, respectively. Each condition was assessed in quadruplicate wells, and all experiments were performed in three independent biological replicates. Detailed information on the drugs and reagents used is provided in Table S1.

### 2.8. Statistical Analysis

All statistical analyses were performed using R (version 4.5.0). For TCGA gene expression analyses, differences between normal and LUAD tissues were assessed using Welch’s two-sample *t*-test. Other comparisons between two groups were performed using Student’s *t*-test or the Mann–Whitney *U* test, as appropriate. Comparisons among multiple groups were performed using a one-way analysis of variance (ANOVA) followed by Tukey’s post hoc test. Survival curves were generated using the Kaplan–Meier method and compared using the log-rank test. For the analyses of the 10 candidate genes, *p*-values from gene expression and survival analyses were adjusted separately for multiple comparisons using the Benjamini–Hochberg FDR method. A *p*-value < 0.05 was considered statistically significant; for multiple-testing analyses, an FDR-adjusted *p*-value < 0.05 was considered significant.

## 3. Results

### 3.1. Expression of miR-126-5p and miR-126-3p in LUAD Patients with EGFR Mutation by TCGA Analysis

**Figure 1:**
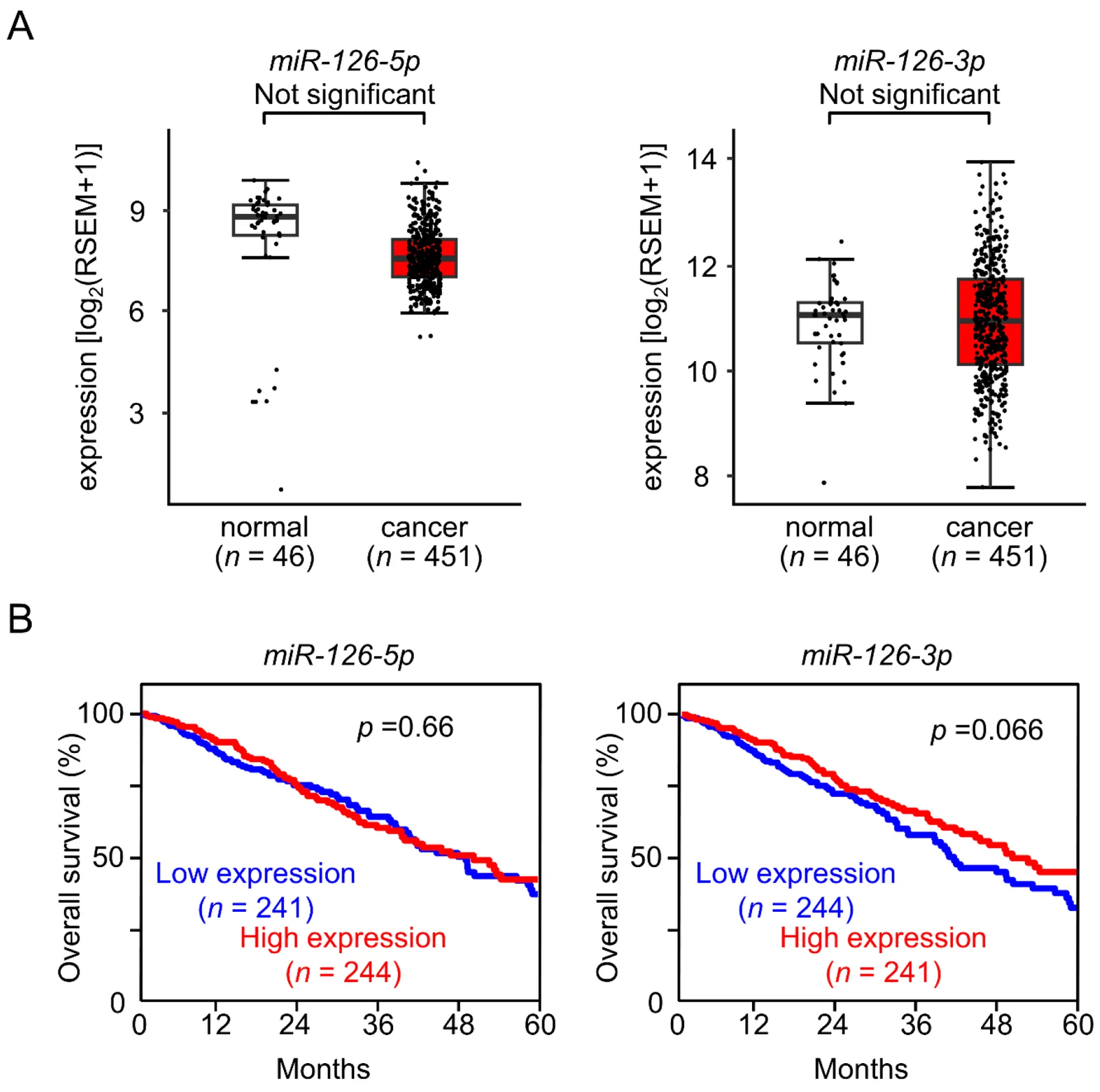
The expression levels and Kaplan–Meier analysis of survival based on miRNA (*miR-126-5p* and *miR-126-3p*) in lung cancer patients using the TCGA database. (**A**) Expression levels in patients with LUAD (46 normal and 451 cancer tissue samples). (**B**) The 5-year overall survival rate of patients with LUAD (*n* = 485).

We recently established miRNA expression signatures using clinical specimens from patients with EGFR-mutant LUAD [17]. The corresponding dataset is available in the Gene Expression Omnibus (GEO) under accession number GSE330633. Based on these signatures, we focused on *miR-126-5p* and *miR-126-3p* because both strands were downregulated and showed relatively large fold changes in EGFR-mutant LUAD specimens. We further evaluated their expression and prognostic relevance using The Cancer Genome Atlas (TCGA) dataset. Neither miRNA showed a significant difference in expression between tumor and normal tissues (Figure 1A). In addition, five-year overall survival did not differ significantly between the high- and low-expression groups for either miRNA (Figure 1B).

### 3.2. Antitumor Functions of miR-126-5p and miR-126-3p in LUAD Cell Lines with EGFR Mutation

**Figure 2:**
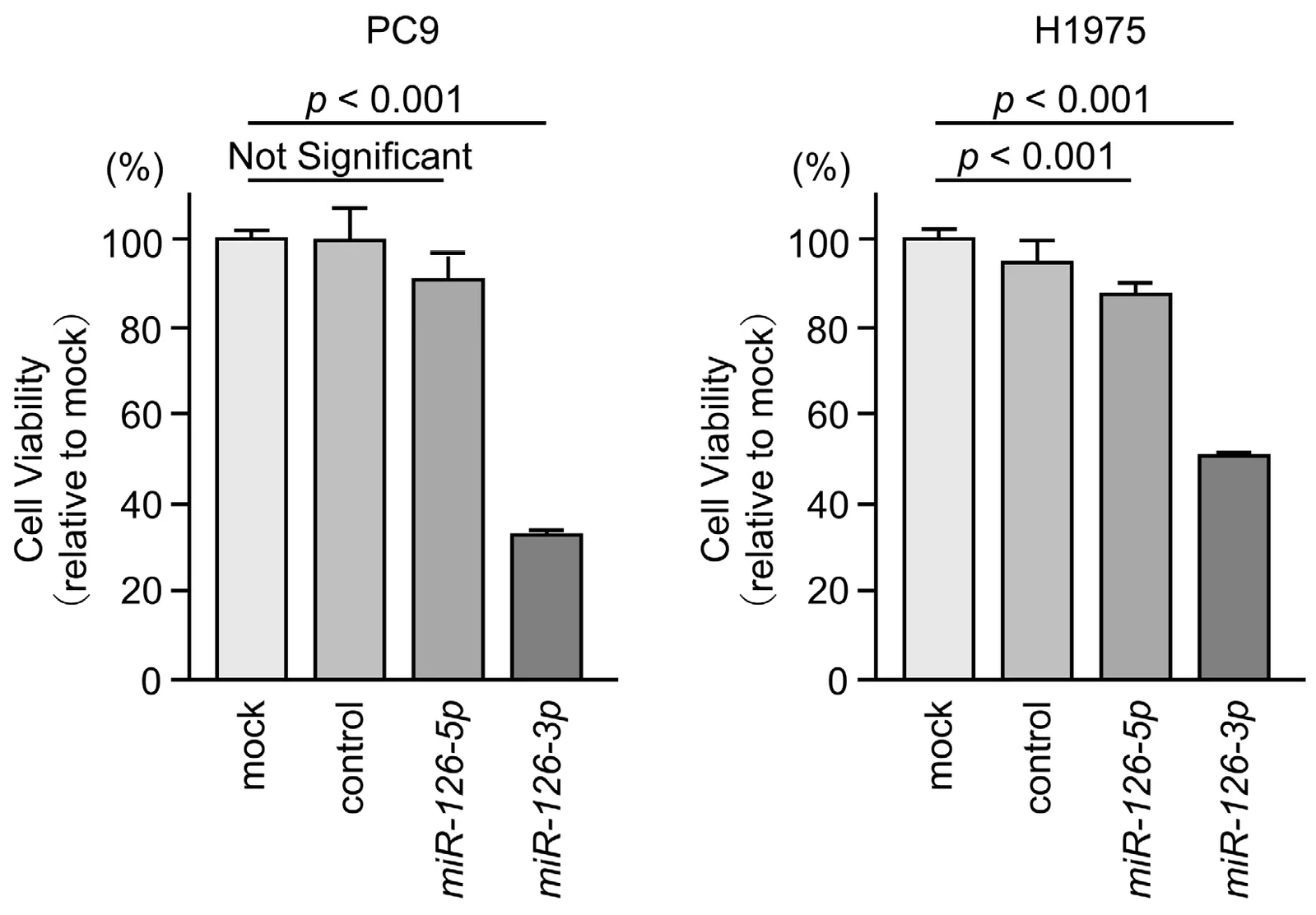
Cell viability of *miR-126-5p* and *miR-126-3p* in PC9 and H1975 cells.

Two cell lines with EGFR mutations (PC9 and H1975) were used for the functional analysis of *miR-126-5p* and *miR-126-3p*. The ectopic expression of *miR-126-3p* significantly suppressed cell viability in both cell lines (Figure 2). Based on these findings, we focused on *miR-126-3p* in the present study.

Cells were transfected with an miRNA precursor (10 nM), negative control miRNA (10 nM), or transfection reagent alone (mock), and cell viability was assessed by CCK-8 assay after 72 h. Values were normalized to mock-transfected cells (100%) and are presented as the mean ± SD from at least three independent experiments. *p*-values were calculated using a one-way ANOVA followed by Tukey’s post hoc test and are shown in each panel.

### 3.3. Identification of Oncogenic Pathways and Therapeutic Targets Regulated by miR-126-3p in LUAD with EGFR Mutation

A search of the TargetScan database revealed that 544 genes contain *miR-126-3p*-binding sequences in their 3′UTR. Using the GeneCodis 4 database, these genes were found to be enriched in the following molecular pathways: Autophagy, the Longevity-regulating pathway, and EGFR tyrosine kinase inhibitor resistance (Table 1).

**Figure 3:**
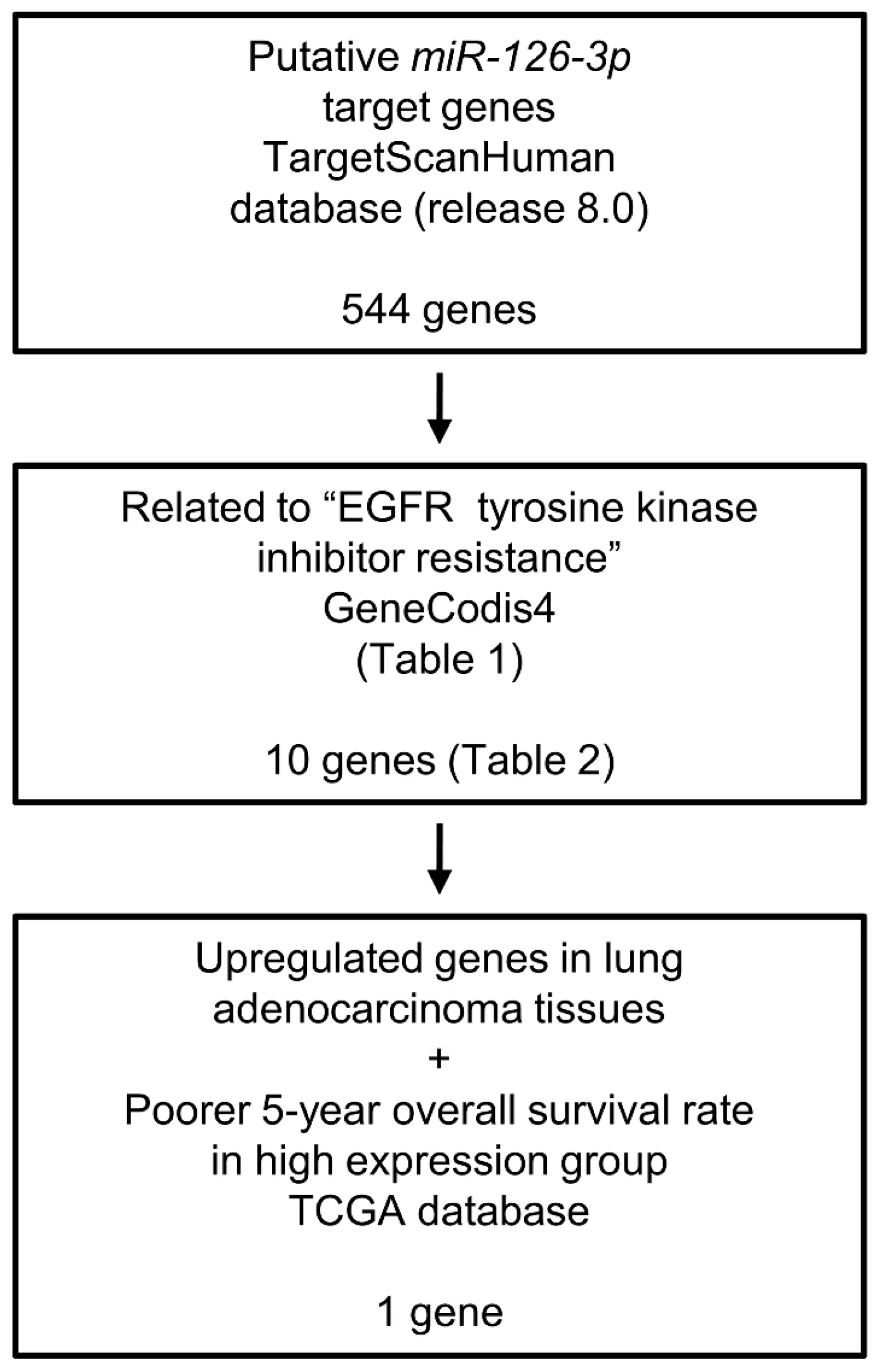
A flowchart for the identification of therapeutic targets regulated by *miR-126-3p* in LUAD with *EGFR* mutations. TargetScan identified 544 predicted *miR-126-3p* target genes, 10 of which were associated with the “EGFR tyrosine kinase inhibitor resistance” pathway. Their predicted *miR-126-3p*-binding sites, expression profiles, and prognostic significance are summarized in Table 2. TCGA-based analysis identified one gene that was upregulated in LUAD tissues and associated with poor five-year overall survival.

Next, we focused on the 10 genes involved in “EGFR tyrosine kinase inhibitor resistance” pathways and narrowed down this list to identify candidate therapeutic targets for *EGFR*-mutant LUAD. The overall workflow for candidate selection is summarized in Figure 3.

**Figure 4:**
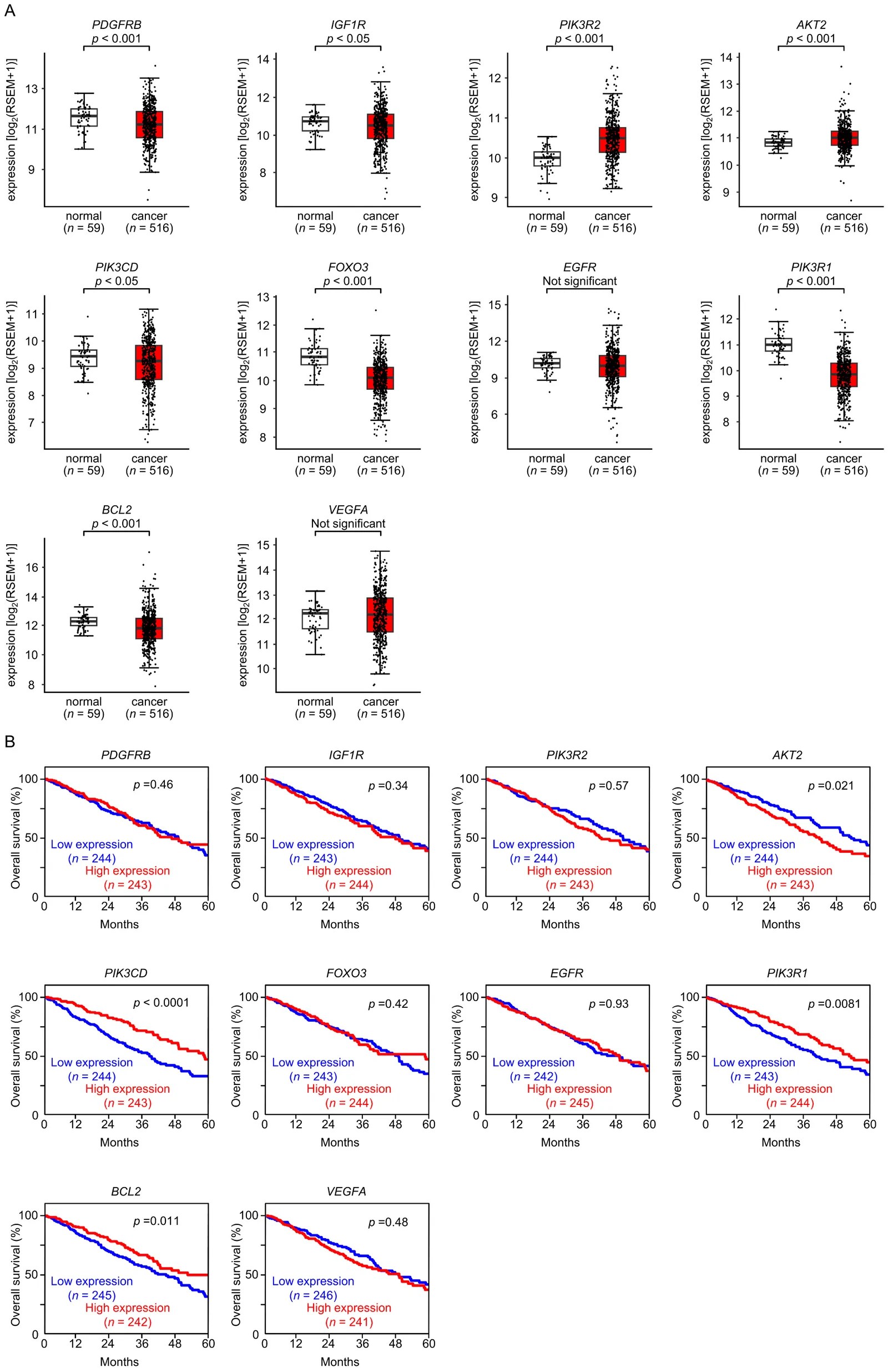
TCGA-based analysis of 10 candidate genes in lung adenocarcinoma. (**A**) Expression levels of 10 candidate genes in LUAD tumor tissues (*n* = 516) and normal lung tissues (*n* = 59). (**B**) Kaplan–Meier analysis of five-year overall survival in high- and low-expression groups for each candidate gene in patients with LUAD (*n* = 487).

When we examined the expression of these genes using TCGA, we found that the expression of two genes (*AKT2* and *PIK3R2*) was upregulated in LUAD tissues (Figure 4A and Table 2). Furthermore, the high expression level of one gene (*AKT2*) was associated with a poor prognosis in patients with LUAD (Figure 4B and Table 2). After FDR correction, the differential expression of *AKT2* and *PIK3R2* remained significant, whereas the prognostic association of *AKT2* did not (FDR = 0.071).

### 3.4. Regulation of 10 Target Genes in miR-126-3p-Transfected LUAD Cells

**Figure 5:**
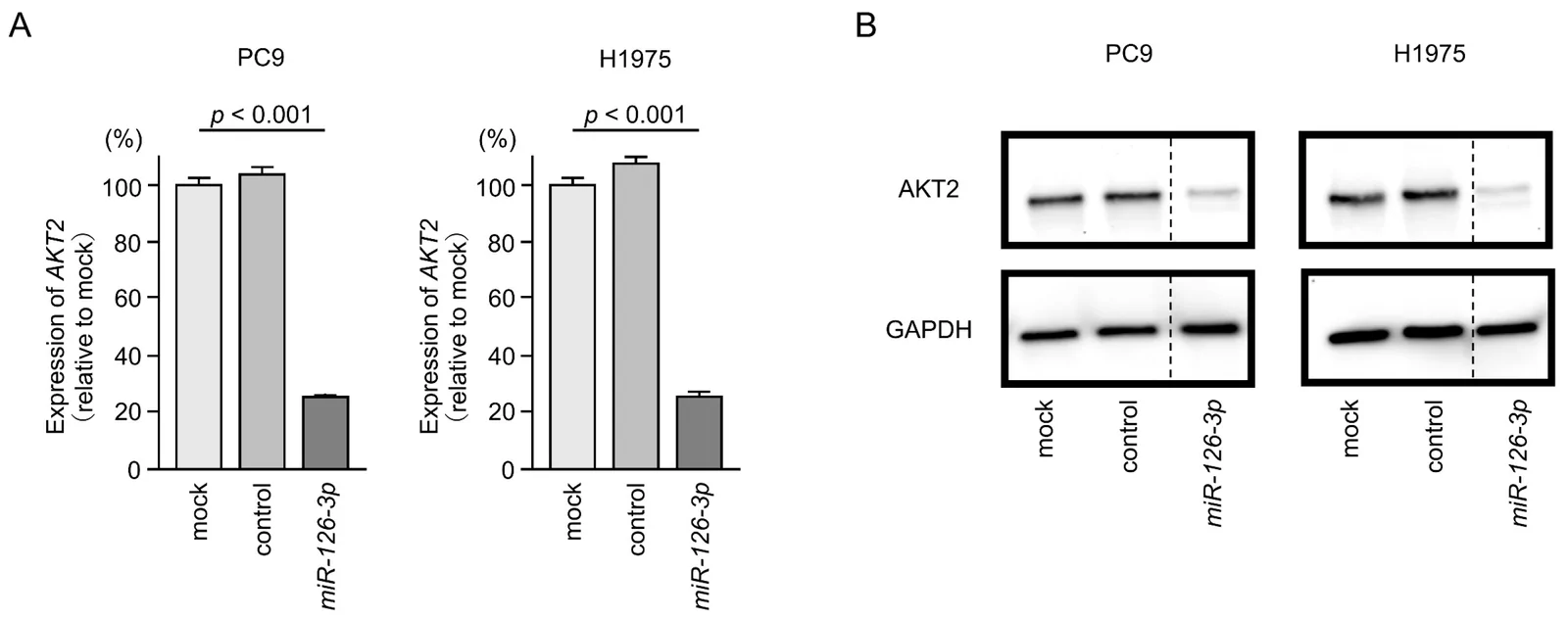
Regulation of *AKT2* by *miR-126-3p* in EGFR-mutated LUAD cells. (**A**) Expression of *AKT2* was significantly reduced in *miR-126-3p*-transfected PC9 and H1975 cells compared with parental cells. Glyceraldehyde-3-phosphate dehydrogenase (*GAPDH*) was used as internal control. (**B**) Expression of AKT2 protein levels was markedly reduced in *miR-126-3p*-transfected PC9 and H1975 cells. Protein samples were collected 72 h after *miR-126-3p* transfection and quantified by Western blotting. GAPDH was used as internal control. Nonadjacent regions of same blot are separated by dashed lines.

To identify genes regulated by *miR-126-3p*, we examined the expression of 10 candidate target genes after *miR-126-3p* transfection in LUAD cells. *miR-126-3p* significantly reduced *PIK3R2*, *AKT2*, and *EGFR* expression in both PC9 and H1975 cells (Figure 5 and Figures S1 and S2). Western blot analysis further confirmed that *miR-126-3p* reduced AKT2 protein expression. Full-size images of Western blots are shown in Figure S3. These findings suggest that *miR-126-3p* regulates *AKT2* at both the mRNA and protein levels in EGFR-mutant LUAD cells.

### 3.5. Effect of Combined Capivasertib (AKT Inhibitor) and Osimertinib (EGFR Inhibitor) on LUAD Cells with EGFR Mutations

Among the *miR-126-3p* target genes identified, we focused on *AKT2*, which is closely involved in the molecular pathogenesis of *EGFR*-mutant LUAD. More recently, capivasertib, a molecularly targeted drug that inhibits *AKT*, has been approved for hormone receptor-positive, HER2-negative breast cancer. Therefore, we investigated the effectiveness of combination therapy with capivasertib and osimertinib in LUAD cell lines with *EGFR* mutations (PC9 and H1975).

**Figure 6:**
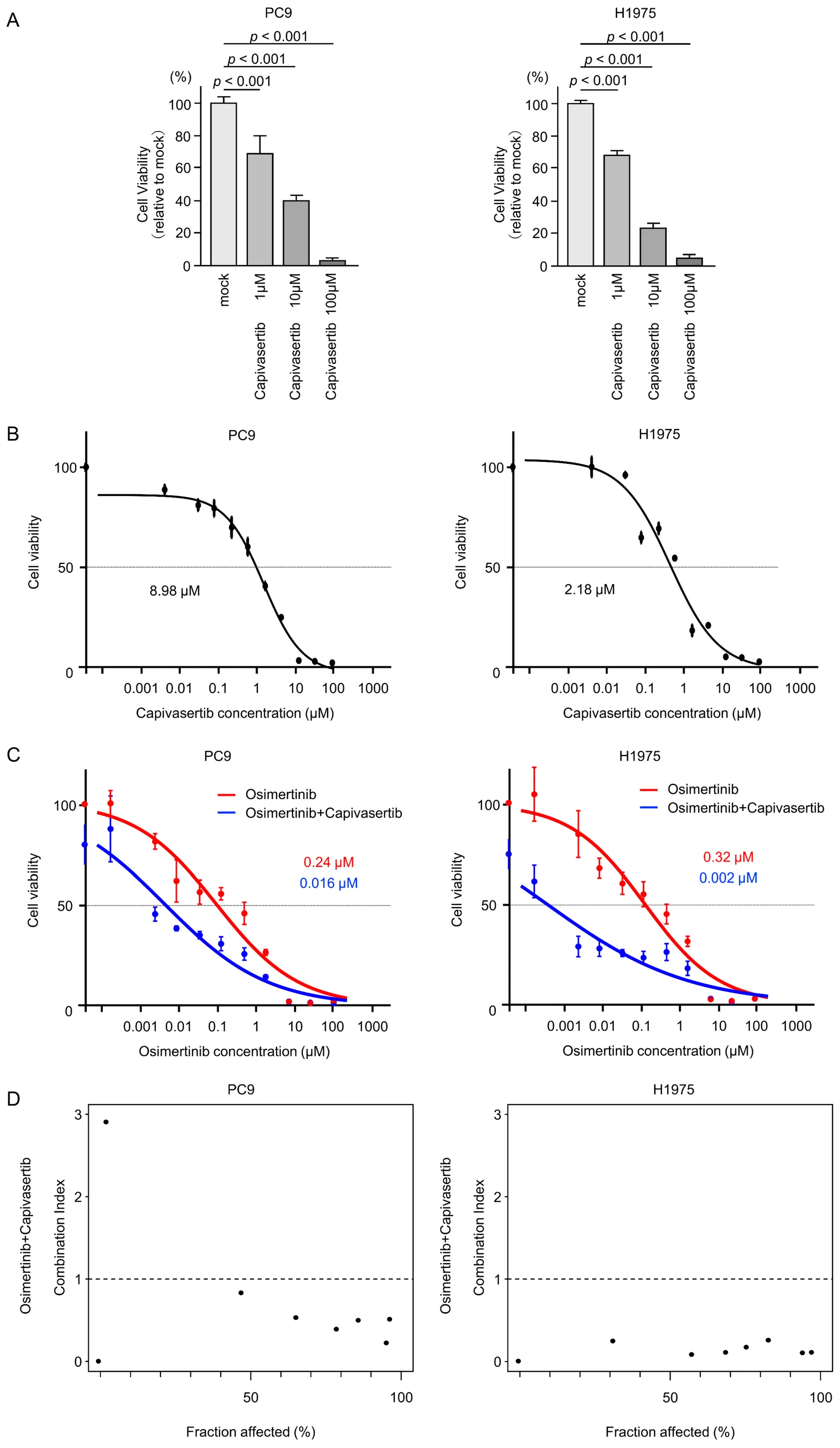
Effects of combination therapy comprising osimertinib and capivasertib on *EGFR*-mutant LUAD cell lines (PC9 and H1975). (**A**) Effect of AKT inhibitor capivasertib on PC9 and H1975 cell viability. Viability was assessed by CCK-8 assay at 72 h with capivasertib in *EGFR*-mutant LUAD cells. (**B**) IC50 of capivasertib in PC9 and H1975 cells, determined based on 72 h CCK-8 assay. Cell viability of *EGFR*-mutant LUAD cells was inhibited by capivasertib. (**C**) Effects of low-dose capivasertib on osimertinib sensitivity in PC9 and H1975 cells, assessed at 72 h. LUAD cells exhibited increased sensitivity to osimertinib when co-treated with 1µM capivasertib. (**D**) Synergistic effect of osimertinib and capivasertib on PC9 and H1975 cells. Synergistic interactions between osimertinib and capivasertib were evaluated using Chou–Talalay method at 72 h.

The antitumor effects of capivasertib were evaluated in PC9 and H1975 cells. Capivasertib inhibited cell viability in a dose-dependent manner in both cell lines (Figure 6A). The IC50 value of capivasertib was 8.98 µM in PC9 cells and 2.18 µM in H1975 cells (Figure 6B).

Next, the effects of capivasertib combined with osimertinib were evaluated. Capivasertib was used at a fixed concentration of 1 µM, corresponding to the IC20, with varying concentrations of osimertinib. The combination treatment markedly reduced the IC50 values, from 0.24 to 0.016 µM in PC9 cells and from 0.32 to 0.002 µM in H1975 cells (Figure 6C). Furthermore, the effect of the combination therapy was assessed using the Chou–Talalay method. At a fixed ratio of 1:10 of osimertinib to capivasertib, the combination index (CI) at Fa = 0.5 was 0.61 in PC9 cells and 0.14 in H1975 cells, indicating a synergistic effect (Figure 6D). These results suggest that the inhibition of *AKT* signaling enhances the sensitivity of *EGFR*-mutant LUAD cells to osimertinib.

## 4. Discussion

Currently, the first-line treatment for patients with advanced LUAD harboring *EGFR* mutations is EGFR-TKIs. Furthermore, therapies for use in combination with EGFR-TKIs have been developed, providing more personalized treatment for LUAD patients [20,21]. However, lung cancer cells frequently acquire drug resistance during EGFR-TKI treatment. Previous studies investigating the molecular mechanisms of EGFR-TKI resistance have identified new *EGFR* mutations, mutations in other driver genes, and the activation of cancer pathways other than the *EGFR* pathway.

Osimertinib, the first third-generation EGFR-TKI, is the leading EGFR-TKI today because it selectively inhibits both EGFR-TKI-sensitive mutations and the T790M resistance mutation [22]. However, the effectiveness of osimertinib decreases during treatment due to the emergence of new *EGFR* mutations (e.g., C797S, L718Q, and G724S), as well as the activation of cancer pathways via *MET* amplification, *HER2* amplification, and *KRAS* mutations [23].

Because a single miRNA controls the expression of a vast number of protein-coding genes, cellular RNA networks can be explored by starting with miRNAs. We previously reported oncogenic molecular pathways regulated by tumor-suppressive miRNAs within lung cancer cells [16,17,24]. To explore those pathways activated in *EGFR*-mutant LUAD, we previously generated miRNA expression signatures using cancer tissues derived from LUAD patients with *EGFR* mutations.

Previous reports have shown that *miR-126* (*miR-126-5p* and *miR-126-3p*) regulates various processes essential for cell maintenance, including angiogenesis, inflammation, and fibrosis [25]. In cancer research, the suppression of *miR-126-3p* expression has been reported in various types of cancer, including lung cancer [25,26]. Furthermore, *miR-126-3p* has been shown to regulate several important genes involved in oncogenic pathways, such as the *PI3K/AKT/mTOR* pathway and the *VEGF-A/VEGFR2/MAPK* pathway [27,28]. Recent studies have revealed that AKT2 kinase is a novel direct target of *miR-126-3p* in breast cancer cells. Furthermore, high expression levels of *miR-126-3p* and low expression levels of *AKT2* have been shown to be associated with a favorable long-term prognosis in breast cancer patients [29].

The aberrant activation of the *PI3K* pathway is one of the most critical oncogenic pathways in various types of cancer [30]. *AKT* is a key mediator of the oncogenic functions of *PI3K* and therefore has been investigated by numerous studies as a therapeutic target for various cancers [31]. However, the development of AKT inhibitors has been stalled for many years, and their clinical efficacy is disappointing.

Capivasertib (formerly AZD-5363) is a molecularly targeted drug that targets all three *AKT* isoforms (*AKT1/2/3*) and inhibits cancer signaling activated by mutations in *PIK3CA*, *AKT1*, and *PTEN* [32]. Capivasertib was recently approved as a combination therapy with fulvestrant for hormone receptor-positive, HER2-negative advanced breast cancer with one or more gene mutations in *PIK3CA*, *AKT1*, or *PTEN* [33]. In the CAPItello-291 phase 3 clinical trial, this combination therapy significantly improved the progression-free survival of patients with luminal-type breast cancer [18].

Even osimertinib, one of the strongest EGFR inhibitors, is ineffective against cancer cells that have developed resistance [9,23]. A study examining circulating tumor DNA in patients who had lost response to osimertinib treatment identified gene mutations involved in the *PIK3CA/AKT/PTEN* pathway [34,35]. NSCLC cells with *EGFR* mutations acquired resistance to osimertinib when additional *PIK3CA/AKT/PTEN* gene mutations arose. Importantly, this resistance was resolved with combination therapy comprising osimertinib and the AKT inhibitor capivasertib [35]. These findings indicate that the acquisition of mutations in the *PIK3CA/AKT/PTEN* genes is clinically important as a mechanism of osimertinib resistance.

Based on the miRNA signature derived from *EGFR*-mutant LUAD, we focused on *miR-126-3p*, and through miRNA-based analysis, we arrived at the AKT inhibitor capivasertib. We demonstrated that combined osimertinib and capivasertib synergistically suppressed the cell viability of *EGFR*-mutant LUAD cells. By adapting the strategy to other candidate miRNAs, we may discover new therapeutic targets for combination therapy involving EGFR inhibitors.

## 5. Conclusions

Based on the miRNA expression signature created from tumor tissues harboring *EGFR* mutations, we selected *miR-126-3p* for investigation because it was significantly downregulated in patient tissues. After exploring the pathways and genes regulated by *miR-126-3p*, we focused on the genes involved in the EGFR tyrosine kinase inhibitor resistance pathway. Among these candidate genes, *AKT2* was upregulated in LUAD tissues, and its mRNA and protein expression was reduced by the ectopic expression of *miR-126-3p* in *EGFR*-mutant LUAD cells. Combination therapy with the AKT inhibitor capivasertib and the EGFR inhibitor osimertinib synergistically suppressed the cell viability of *EGFR*-mutant cells. These findings suggest that AKT inhibition with capivasertib may represent a potential therapeutic strategy for enhancing the efficacy of osimertinib in *EGFR*-mutant LUAD.

## Figures and Tables

**Table 1:** Pathway enrichment analysis of predicted *miR-126-3p* target genes.

Description	*p*-Value	False Discovery Rate	Enrichment	Genes
Autophagy	<0.001	<0.001	3.67	*ULK2*, *ATG10*, *IGF1R*, *PIK3C3*, *PIK3R2*, *ATG7*, *MAPK8*, *AKT2*, *CTSB*, *PIK3CD*, *ATG2B*, *IRS1*, *RRAGC*, *VPS33A*, *TSC1*, *PIK3R1*, *BCL2*, *IRS2*
Longevity-regulating pathway	<0.001	0.009	5.00	*IGF1R*, *PIK3R2*, *AKT2*, *PIK3CD*, *IRS1*, *HSPA6*, *FOXO3*, *PIK3R1*, *IRS2*
EGFR tyrosine kinase inhibitor resistance	<0.001	0.009	4.31	*PDGFRB*, *IGF1R*, *PIK3R2*, *AKT2*, *PIK3CD*, *FOXO3*, *EGFR*, *PIK3R1*, *BCL2*, *VEGFA*
Fluid shear stress and atherosclerosis	<0.001	0.015	3.18	*HSP90B1*, *PIK3R2*, *ACVR2B*, *MAPK8*, *GSTO2*, *AKT2*, *PIK3CD*, *SDC1*, *PIAS4*, *SDC2*, *PIK3R1*, *BCL2*, *VEGFA*
Focal adhesion	<0.001	0.015	2.72	*CRKL*, *PDGFRB*, *IGF1R*, *CRK*, *TLN2*, *PIK3R2*, *MAPK8*, *ITGA6*, *AKT2*, *PARVA*, *EMP1*, *PIK3CD*, *EGFR*, *PIK3R1*, *BCL2*, *VEGFA*
Type II diabetes mellitus	<0.001	0.015	5.13	*PIK3R2*, *MAFA*, *MAPK8*, *PIK3CD*, *IRS1*, *PIK3R1*, *IRS2*
Chronic myeloid leukemia	<0.001	0.015	4.03	*CRKL*, *BAK1*, *RUNX1*, *CTBP2*, *CRK*, *PIK3R2*, *AKT2*, *PIK3CD*, *PIK3R1*
*FoxO* signaling pathway	<0.001	0.017	3.11	*AGAP2*, *IGF1R*, *PIK3R2*, *PLK2*, *MAPK8*, *AKT2*, *PIK3CD*, *IRS1*, *FOXO3*, *EGFR*, *PIK3R1*, *IRS2*
Insulin signaling pathway	<0.001	0.020	3.00	*PRKCI*, *CRKL*, *CRK*, *PIK3R2*, *MAPK8*, *AKT2*, *PIK3CD*, *IRS1*, *ACACA*, *TSC1*, *PIK3R1*, *IRS2*
Neurotrophin signaling pathway	<0.001	0.020	3.16	*FRS2*, *CRKL*, *CRK*, *PIK3R2*, *MAPK8*, *AKT2*, *PIK3CD*, *IRS1*, *FOXO3*, *PIK3R1*, *BCL2*
*AMPK* signaling pathway	<0.001	0.021	3.11	*IGF1R*, *PIK3R2*, *AKT2*, *PPP2R1A*, *PIK3CD*, *IRS1*, *FOXO3*, *ACACA*, *TSC1*, *PIK3R1*, *IRS2*
Prolactin signaling pathway	<0.001	0.023	3.88	*TNFSF11*, *PIK3R2*, *TNFRSF11A*, *MAPK8*, *AKT2*, *PIK3CD*, *FOXO3*, *PIK3R1*
Melanoma	0.001	0.024	3.78	*BAK1*, *PDGFRB*, *IGF1R*, *PIK3R2*, *AKT2*, *PIK3CD*, *EGFR*, *PIK3R1*

**Table 2:** Ten predicted *miR-126-3p* target genes in the EGFR tyrosine kinase inhibitor resistance pathway.

Entrez Gene ID	Gene Symbol	Gene Name	Expression in LUAD Tissues	False Discovery Rate	Expression Group Associated with Poor Five-Year Overall Survival *p*-Value	False Discovery Rate
5159	*PDGFRB*	platelet-derived growth factor receptor, beta polypeptide	Downregulated	<0.001	0.46	0.655
5465	*IGF1R*	insulin-like growth factor 1 receptor	Downregulated	0.034	0.34	0.655
5296	*PIK3R2*	phosphoinositide-3-kinase, regulatory subunit 2 (beta)	Upregulated	<0.001	0.57	0.715
208	*AKT2*	AKT serine/threonine kinase 2	Upregulated	<0.001	0.021 (High expression)	0.071
8977	*PIK3CD*	phosphatidylinositol-4,5-bisphosphate 3-kinase catalytic subunit delta	Downregulated	0.019	<0.0001 (Low expression)	<0.001
3821	*FOXO3*	forkhead box O3	Downregulated	<0.001	0.42	0.655
3236	*EGFR*	epidermal growth factor receptor	Not significant	0.098	0.93	0.928
5295	*PIK3R1*	phosphoinositide-3-kinase, regulatory subunit 1 (alpha)	Downregulated	<0.001	0.0081 (Low expression)	0.928
990	*BCL2*	BCL2 apoptosis regulator	Downregulated	<0.001	0.011 (Low expression)	0.055
12680	*VEGFA*	vascular endothelial growth factor A	Not significant	0.098	0.26	0.654

## Data Availability

Publicly available datasets were analyzed in this study. These data can be accessed here: https://www.ncbi.nlm.nih.gov/geo/query/acc.cgi?acc=GSE330633 (accessed on 1 July 2026).

## Supplementary Materials

The following supporting information can be downloaded at https://www.mdpi.com/article/10.3390/genes17091016/s1, Figure S1: Expression levels of 10 genes in *miR-126-3p*-transfected PC9 cells; Figure S2: Expression levels of 10 genes in *miR-126-3p*-transfected H1975 cells; Figure S3: Full-length Western blot images of AKT2 in *miR-126-3p*-transfected PC9 and H1975 cells 72 h after transfection; Table S1: List of reagents and primers used in this study.

## Abbreviations

The following abbreviations are used in this manuscript:

miRNA	microRNA
NSCLC	non-small cell lung cancer
EGFR	epidermal growth factor receptor
LUAD	lung adenocarcinoma
TKIs	tyrosine kinase inhibitors
CCK-8	Cell Counting Kit-8
ANOVA	analysis of variance
FDR	false discovery rate
CI	combination index
GEO	Gene Expression Omnibus
TCGA	The Cancer Genome Atlas
